# Age-related increase of alpha-synuclein oligomers is associated with motor disturbances in L61 transgenic mice

**DOI:** 10.1016/j.neurobiolaging.2021.01.010

**Published:** 2021-01-28

**Authors:** Sahar Roshanbin, Agata Aniszewska, Astrid Gumucio, Eliezer Masliah, Anna Erlandsson, Joakim Bergström, Martin Ingelsson, Sara Ekmark-Lewén

**Affiliations:** a Department of Public Health and Caring Sciences, Uppsala University, Uppsala, Sweden; b Division of Neurosciences, NIA-NIH, Bethesda, USA

**Keywords:** Parkinson’s disease, Dementia with Lewy bodies, Alpha-synuclein, Transgenic mice, Sex differences, Aggregation, Oligomers, Thy-1, Age progression

## Abstract

The pathogenesis of Parkinson’s disease involves fibrillization and deposition of alpha-synuclein (α-syn) into Lewy bodies. Accumulating evidence suggests that α-syn oligomers are particularly neurotoxic. Transgenic (tg) mice overexpressing wild-type human α-syn under the Thy-1 promoter (L61) reproduce many Parkinson’s disease features, but the pathogenetic relevance of α-syn oligomers in this mouse model has not been studied in detail. Here, we report an age progressive increase of α-syn oligomers in the brain of L61 tg mice. Interestingly, more profound motor symptoms were observed in animals with higher levels of membrane-bound oligomers. As this tg model is X-linked, we also performed subset analyses, indicating that both sexes display a similar age-related increase in α-syn oligomers. However, compared with females, males featured increased brain levels of oligomers from an earlier age, in addition to a more severe behavioral phenotype with hyperactivity and thigmotaxis in the open field test. Taken together, our data indicate that α-syn oligomers are central to the development of brain pathology and behavioral deficits in the L61 tg α-syn mouse model.

## 1. Introduction

The progressive accumulation of α-synuclein (α-syn) into insoluble fibrillar inclusions known as Lewy bodies and Lewy neurites is central to the pathogenesis of Parkinson’s disease (PD) and other α-synucleinopathies. Alpha-synuclein is a small, evolutionarily conserved protein and its gene, *SNCA*, is abundantly expressed in the central nervous system. It is enriched in presynaptic terminals (Maroteaux et al., 1988; Kahle & et al., 2000; Yang & et al., 2010) and mediates its cellular function by interacting with synaptic vesicle membranes (Burre, 2015). Among proposed functions, α-syn is believed to support the assembly of soluble N-ethylmaleimide-sensitive factor attachment protein receptor complex (Burre & et al., 2010) in the presynapse and thereby regulates vesicular packaging and trafficking (Kahle & et al., 2000; Fortin & et al., 2004; Ross & et al., 2008). Importantly, α-syn is conformationally flexible and has been found to adopt different structures. On interaction with membranes, cytosolic α-syn shifts from a natively unfolded and unstructured state to a membrane-bound, alpha-helical structure (Burré et al., 2014). However, the intrinsic properties of α-syn allow for a shift of its conformation into a pathogenic state, with self-assembly into β-sheet–rich structures that promote the formation of oligomers, protofibrils, and eventually fibrils.

Soluble α-syn oligomers have been shown to be toxic both in vitro (Danzer & et al., 2007; Kayed & et al., 2003; van Rooijen et al., 2008) and in vivo (Karpinar & et al., 2009; Winner & et al., 2011) and are thus believed to play a central pathophysiological role in α-synucleinopathies (Sharon & et al., 2003; Kramer and Schulz-Schaeffer, 2007; Paleologou & et al., 2009). The neurotoxicity of soluble α-syn aggregates can be attributed to disruption of associated membranes and thereby disturbances in synaptic integrity.

Animal models expressing human α-syn have become useful tools for the study of α-synucleinopathies and to assess the pathogenic role of oligomers. For example, rats transduced with lentiviral vectors expressing the artificial oligomerization-prone α-syn mutants *E35 K* and *E57 K* into substantia nigra were shown to have a higher degree of dopaminergic cell loss in mesencephalon compared with animals transduced with wild type (wt) α-syn and various other α-syn mutants (Winner & et al., 2011). Moreover, we have previously investigated mice overexpressing the disease-causing *A30P* mutation ((Thy-1)-h[A30P] mice) and found that spinal cord levels of α-syn oligomers are increased in animals with a more severe motor phenotype—including paralysis of hind limbs, loss of coordination, and staggering gait (Lindstrom & et al., 2014). In addition, we demonstrated that immunotherapy with an oligomer selective α-syn monoclonal antibody resulted in lower oligomer levels in the spinal cord of such mice (Lindstrom & et al., 2014).

However, both patients with *SNCA* duplication (Ibáñez & et al., 2004) and triplication (Singleton & et al., 2003) as well as the vast majority of sporadic PD patients develop brain pathology based on wt α-syn. Thus, α-syn can start to aggregate also in its nonmutated form, potentially because of increased levels or as a result of post-translational modifications, such as phosphorylation at serine 129 (S129) and serine 87 (S87) (Anderson & et al., 2006).

Mice overexpressing wt human α-syn under the Thy-1 promoter (L61 mice) have previously been shown to reproduce several features of PD (Rockenstein & et al., 2002), including robust α-syn brain pathology, loss of dopaminergic terminals in basal ganglia, and motor impairments (Lam & et al., 2011). However, as no studies have investigated the pathogenic role of α-syn oligomer in L61 mice, we decided to examine the relationship between α-syn oligomerization and the development of behavioral dysfunction in this mouse model.

## 2. Methods

### 2.1. Animals

All experiments were approved by the Uppsala Animal Ethics Committee and the use of mice was conducted in accordance with the EU directive 2010/63/EU for animal experiments.

The L61 transgenic (tg) mice were developed in the laboratory of Eliezer Masliah at UCSD on a C57BL6/DBA2 background (Rockenstein & et al., 2002). Mice were bred by crossing C57BL6/DBA2 males with heterozygous L61 females on a C57BL6/DBA2 background. All experiments were performed on 3-, 6-, 9-, and 12-month-old L61 tg mice as well as age- and sex-matched nontransgenic (ntg) littermates (n = 12–14/group for L61 tg mice for biochemical analyses and n = 13–22 for L61 tg mice/n = 4–10 for ntg controls for behavioral analyses). In total, 99 mice were included in the study. For the survival analysis, data were collected from 200 mice in our breeding colony (n = 50/group). Animals were housed in groups of maximum 5 mice in open cages on a 12:12 hour constant light cycle in a temperature- and humidity-controlled room and were given food and water *ad libitum*.

### 2.2. Brain tissue preparation

Mice were transcardially perfused with 0.9% NaCl, after which brains were isolated and hemispheres separated. Right hemispheres were postfixed in 4% PFA solution for 24 hours and transferred to 70% ethanol solution followed by paraffin embedding and cutting into 10 μm sections. The left hemispheres without olfactory bulb, cerebellum, and brain stem were snap frozen on dry ice and stored at −70° C until further biochemical analyses.

### 2.3. Protein extraction

To fractionate α-syn aggregates based on their solubility, we performed sequential protein extraction. Immediately before extraction, the left hemispheres were homogenized with Precellys (Bertin Instruments), with ice-cold tris-buffered saline (TBS) at a 1:5 weight: volume ratio with protease inhibitors (cOmplete, Mini, EDTA-free Protease Inhibitor Cocktail, Roche, Mannheim, Germany, 04693159001). Next, we sequentially extracted TBS, TBS-T (0.5% of Triton X-100), and formic acid (FA, 70%) fractions for extraction of the following α-syn species: monomers and lower-order oligomers (TBS fraction), membrane associated, and higher-older oligomers (TBS-T fraction) and insoluble fibrils (FA fraction).

The same amount of TBS homogenate was taken from each sample and mixed with 1X volume of fresh TBS with protease inhibitors. Next, samples were vortexed for 10 seconds and spun down for 1 hour at 16.000 g at 4° C. The supernatant (TBS soluble fraction) was collected, and the pellet was washed with 500 μL of fresh TBS before being resuspended in TBS-T 0.5% and spun down for 1 hour at 16.000 g at 4° C. The supernatant (TBS-T soluble fraction) was collected, and the pellet was washed with 500 μL of fresh TBS before resuspension in 70% FA. After resuspension, samples were spun down for 1 hour at 100.000 g at 4° C, and the supernatant was collected. All aliquots were stored in −70° C until further analyses. The protein extraction procedure is summarized in Supplementary Fig. 1.

### 2.4. Measurement of α-syn levels

The levels of α-syn species in TBS, TBS-T, and FA soluble fractions were measured by 2 different sandwich enzyme-linked immunosorbent assay (ELISAs). First, to detect oligomeric α-syn, we modified a recently developed sandwich ELISA that utilizes the rabbit monoclonal antibody MJFR14-6-4-2. This antibody was validated as a reliable tool for specific detection of α-syn oligomers in detergent-treated brain tissue samples as it is conformation selective, aggregate dependent, and does not detect monomeric α-syn (Lassen & et al., 2018). To further ensure that no α-syn monomers were detected, we used MJFR14-6-4-2 as the coating antibody and biotinylated MJFR14-6-4-2 as the detection antibody (for details see Table 1). Total α-syn levels were detected using MJFR1 and Syn1 antibodies as coating and detection antibodies, respectively.

Briefly, 96-well plates were coated with antibodies designated for each assay and incubated overnight at 4° C. Subsequent steps were performed at room temperature. Plates were blocked for 2 hours in 1% BSA blocking buffer. Next, TBS and TBS-T soluble protein fractions were diluted in 0.1% BSA incubation buffer with 0.05% Tween 20, added and incubated for 2 hours before detection. All coating and detection antibodies used in ELISAs are listed in Supplementary Table 1. Because of high acidity of the 70% FA solution, the FA fraction samples were neutralized with Trizma buffer mix before α-syn-level measurement.

Standard curves utilizing several dilutions of α-syn oligomers (prepared as described in (Nasstrom & et al., 2009)) or recombinant monomeric α-syn were used to determine the concentration of α-syn in brain extracts.

### 2.5. Immunohistochemistry

Immunohistochemistry (IHC) was performed on 10 μm paraffin sections. Before the staining procedure, sections were deparaffinized by incubations in xylene (2 × 10 minutes) and rehydration in ethanol gradient (100% x2, 96%, and 70%, 10 minutes for each incubation) followed by 2 × 10 minutes washing in tap water. Next, the epitope retrieval procedure was performed. Sections were submerged in 25 mM sodium citrate (pH 6.0), brought to boil and then incubated for 35 minutes until reaching room temperature. Subsequently, the sections were washed with PBS (pH 7.3) and stained for the various proteins.

To detect α-syn, α-syn phosphorylated on serine 129 (p129 α-syn), as well as inflammatory markers, sections were blocked in 5% normal goat serum in PBS-T for 30 minutes or treated with M.O.M. kit (Vector Laboratories, Burlingame, CA, USA, BMK-2202) as per manufacturer’s instructions, followed by overnight incubation at 4° C with mouse or rabbit primary antibodies; anti-human α-syn, clone 211 (1:250, Santa Cruz Biotechnology, Santa Cruz, CA, USA), anti-α-syn phospho-Ser129 EP1536Y (1:250, Abcam, Cambridge, UK, ab51253), anti-GFAP (1:500, DAKO, Denmark, Z0334), and anti-Iba1 (1:500, WAKO chemicals, USA, 019–19,741).

For all stainings, sections were thoroughly washed and incubated for 30 minutes with biotinylated goat anti-rabbit IgG (H + L) (Vector, BA-1000) or goat anti-mouse IgG (Vector, BMK-2202), after which they were washed and incubated for 30 minutes with horseradish peroxidase-conjugated streptavidin (Mabtech AB, Stockholm, Sweden, 3310–9) (all diluted 1:250 in PBS). Antibody binding was visualized with NovaRED Chromogen Peroxidase Substrate kit (Vector, SK-4800). Stained sections were dehydrated and fixed in ethanol gradient (70%–100%) and xylene, mounted with Pertex (Histolab, Gothenburg, Sweden, 00,840) and left to dry overnight.

All pictures were acquired using a Nikon Eclipse 80i microscope and NIS-Elements BR 4. 20.00 software.

### 2.6. Assessment of astrocyte activation

A sandwich ELISA was used to determine the levels of astrocyte activation marker glial fibrillary acidic protein (GFAP). The immunohistochemistry was performed as described previously. Coating and detection antibodies used are listed in Table 1. Relative standard optical density values were used as indicators of astrocyte hyperactivity and compared between L61 tg mice from different age groups. Because GFAP was primarily detected in the TBS-T soluble protein extracts, only this fraction was included in the analyses.

For all ELISAs, the antibody binding was visualized using K-Blue Aqueous TMB substrate (Neogen Corporation, Lansing, MI, USA, 331177) and 1 M sulfuric acid was used to stop the colorimetric reaction at appropriate time points. The results were read at 450 nm on Tecan Infinite M200 PRO reader (Tecan Group Ltd, Männedorf, Switzerland). Unless stated otherwise, all experiments were repeated 3 times, using 6–10 tg mice in each age/sex group as well as age- and sex-matched ntg mice as negative controls.

### 2.7. Behavioral testing

Male and female heterozygous L61 tg mice were tested for overt motor symptoms, hind limb clasping, and open field behavior, as well as checked for signs of visible motor symptoms. Before behavioral testing, body weight was measured. To check for visible motor impairments, each mouse was transferred to a clean cage for a 2–3 minutes observation of visible gait problems including unsteady movement and staggering gait and scored as: 0 = no visible motor impairments or 1 = visible motor impairments. The frequency of observed acute stress response to the novel environment was also registered as 1 = no/low stress response, 2 = stressed, and 3 = highly stressed, where the low stress response referred to the ntg mice behavior in the novel cage, including exploratory behaviors as sniffing, rearing, and grooming, while stressed mice were moving fast in the cage and showed freezing behavior. In addition, the highly stressed mice were difficult to pick up from the cage by the researcher, as they showed defensive behavior and tried to escape from the cage.

Hind limb clasping, a marker of disease progression in a number of mouse models of neurodegeneration, was measured in accordance with the following scale: 0 = no clasping, 1 = one leg clasping, and 2 = both legs clasping. The mouse was lifted by the tail near its base for a few seconds, and the procedure was repeated after 5 minutes. The lowest score from the 2 repeats was used for the analysis. The open field test was used to study activity as well as locomotion and all mice were tested for 5 minutes in a 40 × 40 cm square arena. After completed trial, the number of fecal pellets and urine spots was registered. The arena was cleaned with ethanol between every trial. All sessions were videotaped and automatically analyzed using EthoVision XT 14.0 software (Noldus Technology, Wageningen, Netherlands). In addition, the tracks from the movement in the open field test were observed and scored as 1 = cross over profile, 2 = mixed profile, or 3 = thigmotaxis profile. Manual scoring was used to study rearing and grooming behavior with Score 2.2 (Copyright Solids, Uppsala, Sweden).

### 2.8. Statistical analyses

Shapiro-Wilk’s W test was used to determine normal distribution and such data were analyzed with ANOVA followed by a parametric *post hoc* test. In case of non-normally distributed data, appropriate nonparametric tests were instead chosen (Kruskal-Wallis analysis of variance of ranks followed by Mann-Whitney *U* test for group wise comparisons). Unpaired *t*-test was used to analyze the differences between α-syn levels and motor impairments. One-way ANOVA was used to analyze the levels of α-syn in males and females. Statistica 13.4.0.14 software (StatSoft Inc., Tulsa, OK) was used for statistical analyses, and a *p*-value <0.05 was considered statistically significant. Principal component analysis (PCA) (Winner & et al., 2011) was used as a complement to traditional statistics, as it provides an overview of the data set and is a powerful tool to recognize patterns in data, such as outliers and groups. The analyses result in a score plot showing a summary of the relationship among individual mice and a loading plot in which variables important for these relationships can be identified. The 2 plots are complementary and superimposable. SIMCA-P+ 14.0 software (Umetrics AB, Umeå, Sweden) was used for this purpose.

Kaplan-Meier survival curves were generated using Prism 7 software (GraphPad Software Inc, San Diego, CA, USA) and analyzed with log-rank test to evaluate differences between male and female L61 tg mice and respective ntg controls. A *p*-value <0.05 was considered significant, with n = 50 for each group. Animals with a cause of death that was unrelated to the severity of the phenotype (i.e. inclusion in the characterization before the development of severe motor symptoms) were considered censored events in the survival analysis. All data are shown as mean ± SEM unless otherwise stated. GraphPad Prism (v6.07) was used for the preparation of graphs.

## 3. Results

To study age-related progression and distribution of α-syn pathology in L61 tg mice, we analyzed different molecular species of α-syn, neuroinflammation, survival and behavioral deficits in 3-, 6-, 9-, and 12-month-old males and females.

### 3.1. The levels of α-syn oligomers increase with age

To assess the effect of age progression on α-syn aggregation, we measured the levels of α-syn in sequentially extracted protein fractions of decreasing solubility (TBS, TBS-T and FA soluble fractions, for details see Supplementary Fig.1).

**Fig. 1.**
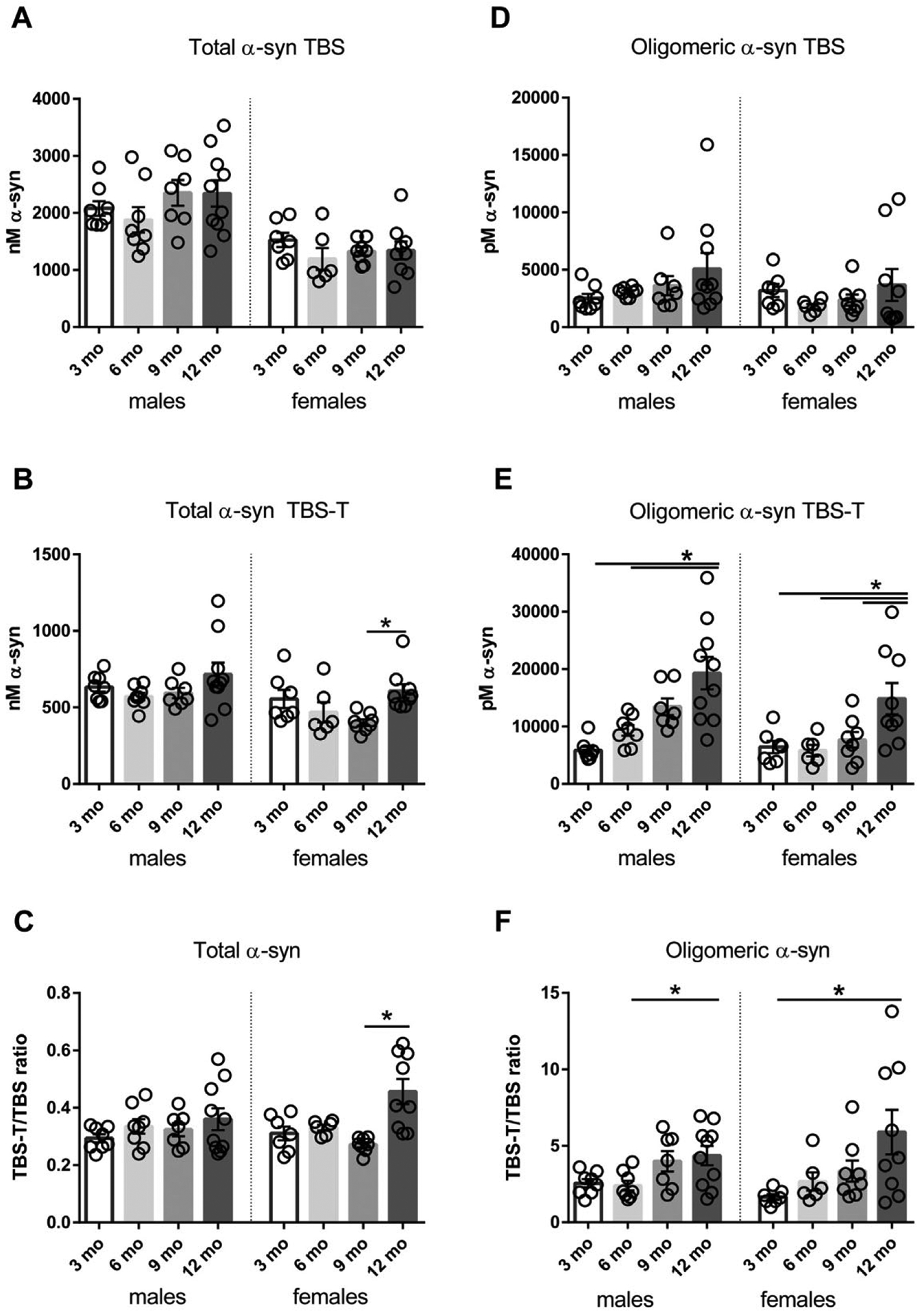
Total and oligomeric α-syn levels in male and female L61 tg mice. Total α-syn levels from TBS (A) and TBS-T soluble fractions (B). Ratio of TBS-T soluble versus TBS soluble total α-syn levels (C). Aggregated α-syn levels from TBS (D) and TBS-T soluble fractions (E). Ratio of TBS-T soluble versus TBS soluble α-syn oligomers (F). Data shown as mean ± SEM, *p < 0.05.

The levels of total α-syn (both TBS and TBS-T soluble) did not differ between 3-, 6-, 9-, and 12-month-old male L61 tg mice (F_(3, 29)_ = 1.025, *p* = 0.395, and F_(3, 29)_ = 1.959, *p* = 0.142, respectively) (Fig. 1A and B). The proportion of TBS-T soluble to TBS soluble total α-syn (Fig. 1C) did not differ between young and aged male mice (F_(3,29)_ = 0.979, *p* = 0.416). Similarly, the levels of TBS soluble total α-syn did not differ between young and old female mice (F_(3, 26)_ = 1.962, *p* = 0.144). Levels of TBS-T soluble α-syn were higher in 12-month- than 9-month-old mice (*p* < 0.05). Consequently, a significant increase was also observed for the ratio of TBS-T to TBS soluble α-syn in female mice (F_(3, 26)_ = 8.511, *p* = 0.0004).

For measurements of soluble α-syn aggregates, we used an ELISA based on the MJFR14-6-4-2 antibody, which detects aggregated α-syn but does not recognize α-syn monomers (Lassen & et al., 2018) (for details see Table 1). No significant age-related changes in the level of TBS soluble α-syn aggregates were observed (F_(3, 29)_ = 1.541, *p* = 0.225 for males and F_(3, 26)_ = 0.799, *p* = 0.505 for females) (Fig 1D). In contrast, the levels of TBS-T soluble α-syn aggregates were increased with age in both males and females (F_(3, 29)_ = 10.07, *p* = 0.0001 and F_(3, 26)_ = 5.190, *p* = 0.0061, respectively) (Fig. 1E). Moreover, as soluble α-syn oligomers are considered particularly neurotoxic, we analyzed the ratio of less soluble species (TBS-T fraction) versus TBS soluble α-syn (Fig. 1F). We found that in both males (F_(3, 29)_ = 3.824, *p* = 0.0201) and females (F_(3, 26)_ = 3.630, *p* = 0.026), the proportion of less soluble α-syn oligomers increased with age.

**Fig. 2.**
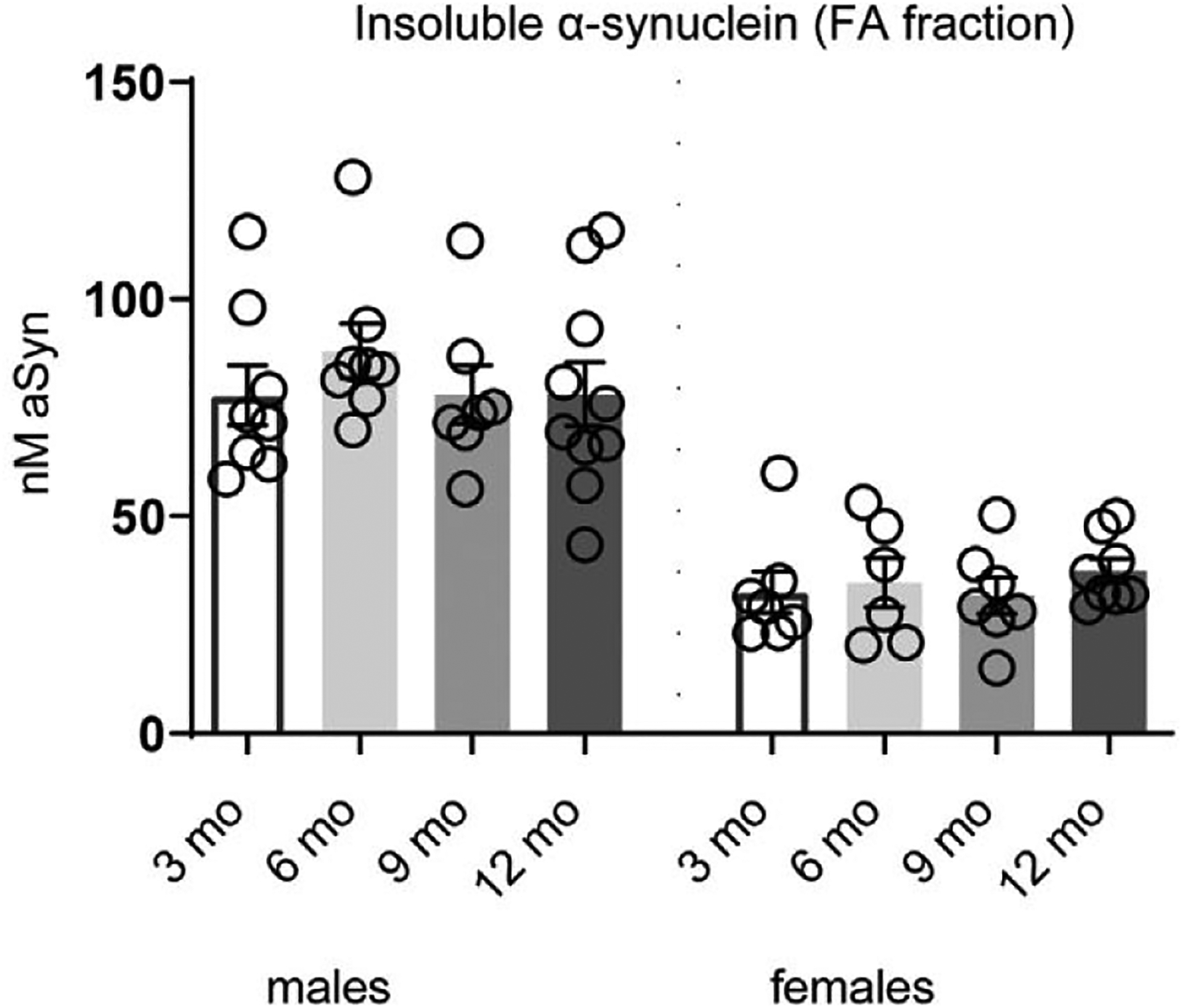
Insoluble α-syn levels in male and female L61 tg mice. Higher levels of total α-syn in formic acid (FA) fraction in male than female L61 tg mice. No age-related changes were detected. Data shown as mean ± SEM.

The levels of insoluble α-syn, measured as the FA protein fraction, did not differ between age groups (F_(3, 29)_ = 0.507, *p* = 0.680 and F_(3, 24)_ = 0.368, *p* = 0.7763 for males and females, respectively), although male mice displayed higher levels (Fig. 2).

### 3.2. Distribution of α-syn pathology in L61 brains

To qualitatively analyze the spatiotemporal pattern of α-syn expression and regional distribution of α-syn pathology, IHC staining using antibodies directed against 2 different α-syn epitopes was performed on brain tissue sections. We stained against p129 α-syn using clone EP1536Y (Delic & et al., 2018) and the epitope 121–125 of human α-syn (clone 211). Based on the oligomer ELISA results, 24 mice (3 males and 3 females from each age group) with the highest levels of α-syn oligomers were analyzed. No sex-related differences in α-syn pathology were observed (data not shown).

**Fig. 3.**
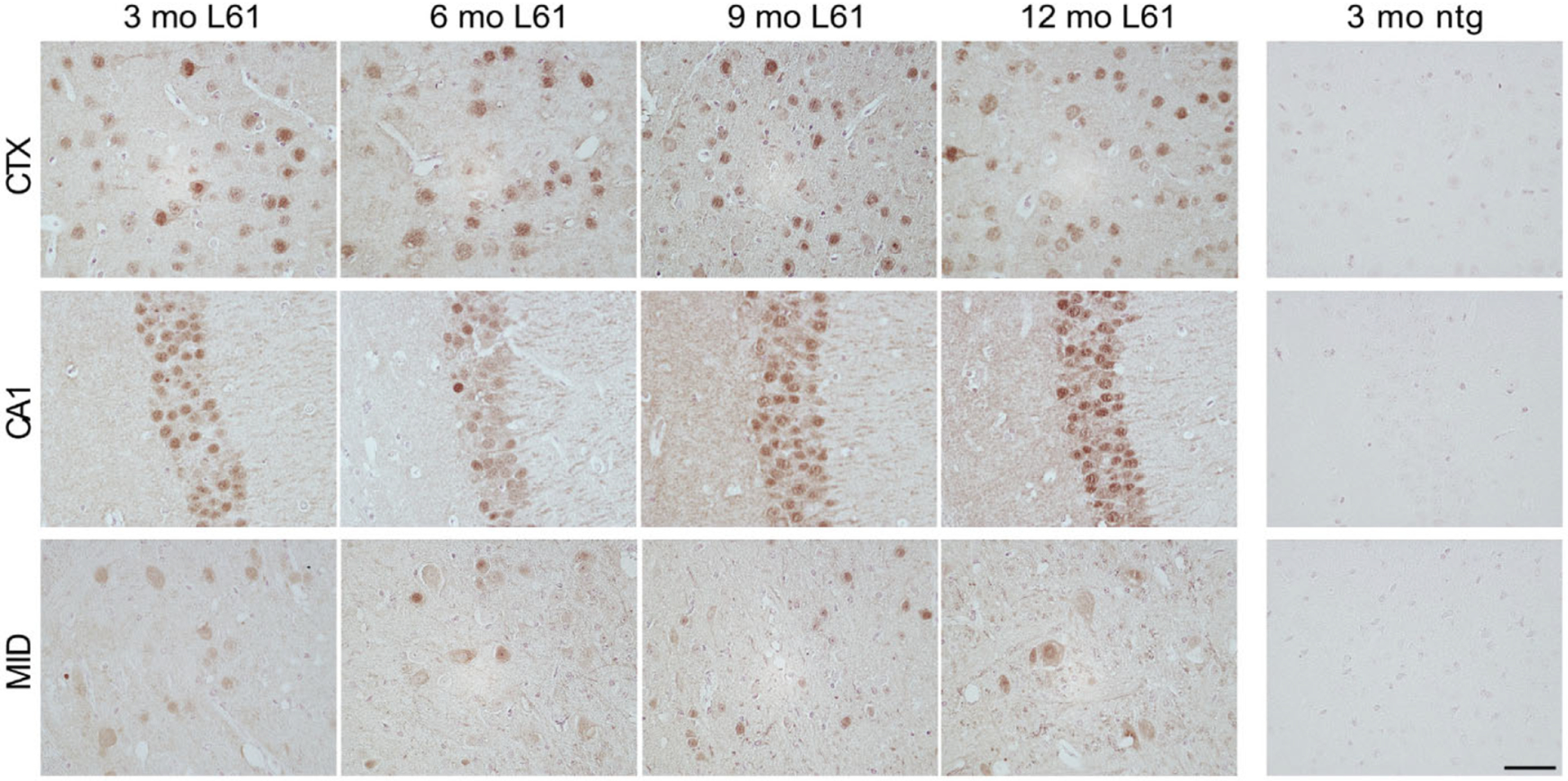
Phosphorylated α-syn pathology in selected areas of the brain. Brain sections from 3-, 6-, 9-, and 12-month-old L61 tg male mice. Immunopositive cells were observed in prefrontal cortex (top panel), hippocampus (middle panel), and midbrain (bottom panel). No immunoreactivity was detected in nontransgenic (ntg) controls. Scale bar: 50 μm, 40x magnification. CTX, cortex; CA1, CA1 region of hippocampus, MID, midbrain.

The extent of α-syn pathology was similar in all age groups, with a profound burden also in the youngest (3 month old) mice. Cells positive for p129 α-syn were present mainly in forebrain, including cerebral cortex (Fig. 3, top panel) and hippocampus, particularly in the CA1 field (Fig. 3, middle panel). Cortical neurons showed a strong p129 α-syn reactivity, especially in cell bodies. Also in hippocampal neurons p129 α-syn was found predominantly in cell bodies (Fig. 3, middle panel), but clear staining of cellular processes was observed also in the CA1 area and in the polymorphic layer of the dentate gyrus (PoDG). More diffuse staining of p129 α-syn was observed in midbrain cells (Fig. 3, bottom panel), where α-syn positive cells were present in the vicinity of substantia nigra, ventral tegmental area (VTA), and the pontine nuclei.

**Fig. 4.**
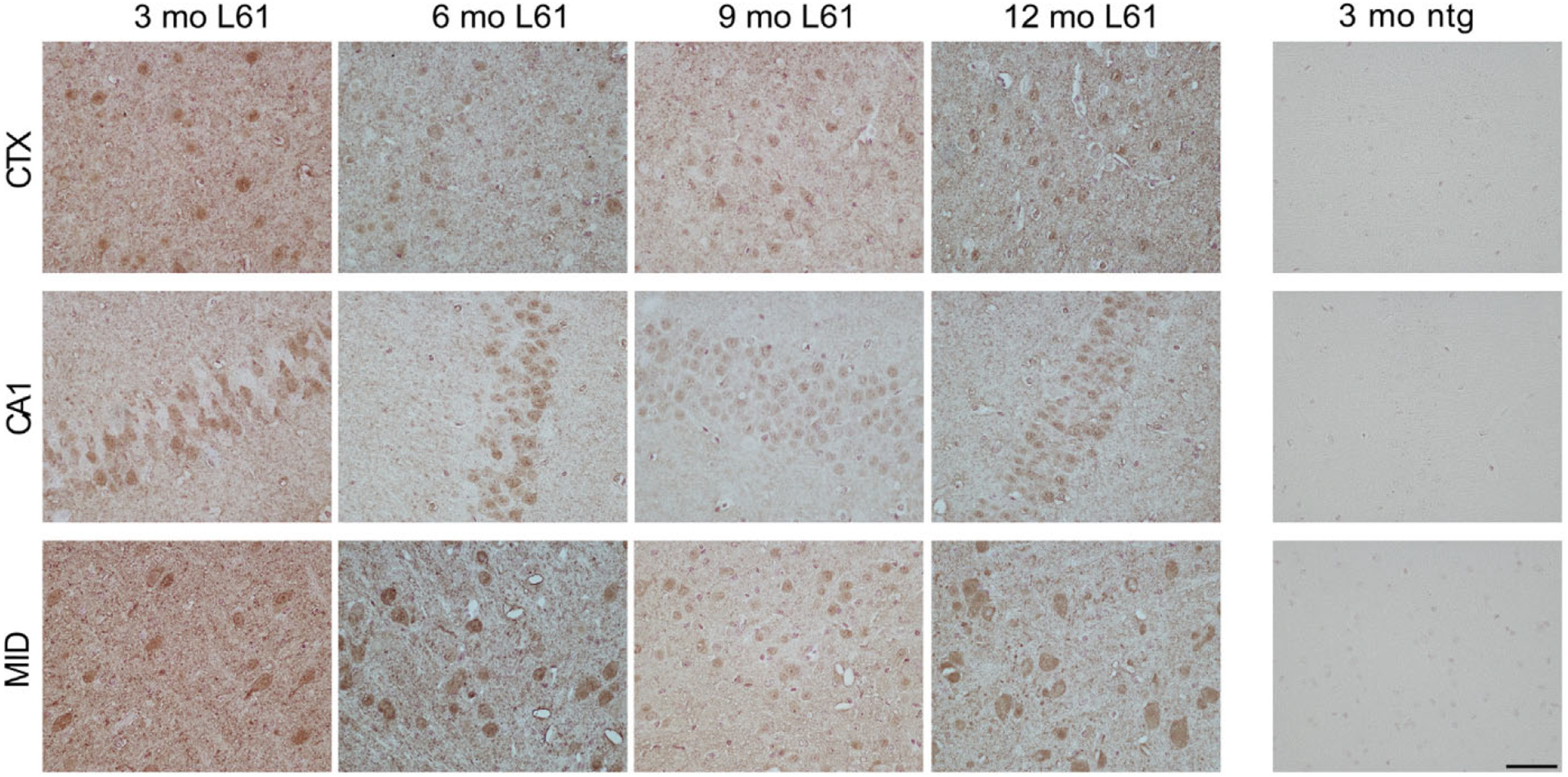
Overall α-syn pathology in selected areas of the brain. Staining with an antibody targeting 121–125 aa of human α-syn on brain sections from 3-, 6-, 9-, and 12-month-old L61 tg male mice. Immunopositive cells were observed in prefrontal cortex (top panel), hippocampus (middle panel), and midbrain (bottom panel). No immunoreactivity was detected in nontransgenic (ntg) controls. Scale bar: 50 μm, 40x magnification. CTX, cortex: CA1, CA1 region of hippocampus: MID, midbrain.

Immunostaining with clone 211 (recognizing epitopes 121–125 (Waxman et al., 2008)) to detect overall human α-syn pathology resulted in a more diffuse cortical staining of both cell bodies and synapses (Fig. 4, top panel). In comparison with the p129 staining, clone 211 positive α-syn was less present in hippocampal cells, where signals were localized in cell bodies as well as in synaptic areas (Fig. 4, middle panel). Whereas p129 α-syn was present mainly in forebrain, clone 211 α-syn staining was most profound in midbrain, including the substantia nigra area (Fig. 4, bottom panel).

### 3.3. Presence of α-syn pathology does not cause excessive gliosis

Considering that α-syn pathology could cause glial cell responses, we also analyzed the extent of astrocytic and microglial activation. Analyses were performed on the 24 mice (3 males and 3 females from each age group) that had been selected for α-syn IHC and on ntg control mice.

**Fig. 5.**
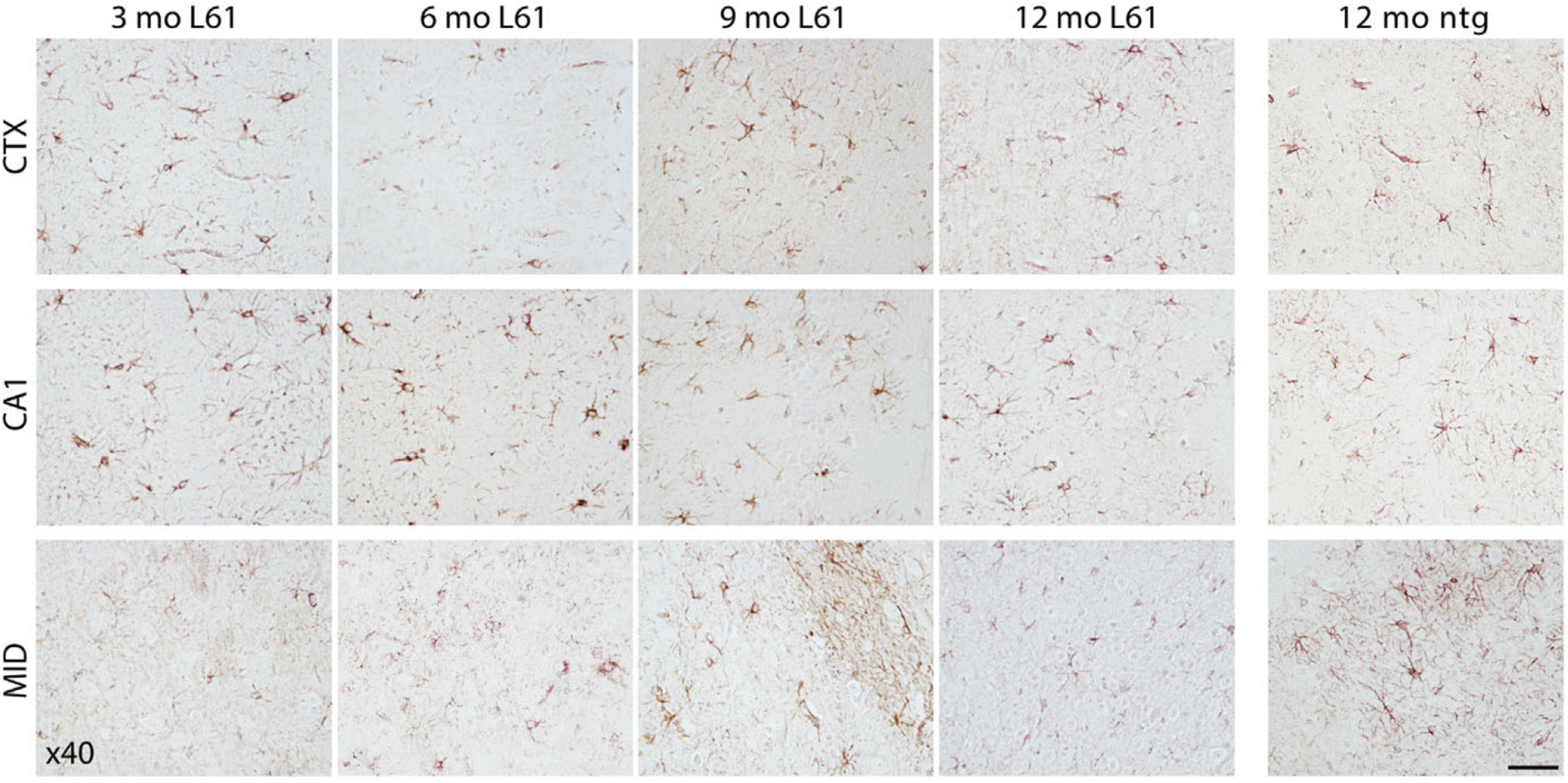
Activated astrocyte morphology and distribution in selected areas of the brain. Immunostaining with an antibody targeting glial fibrillary acidic protein (GFAP). Sections from 3-, 6-, 9-, and 12-month-old L61 tg male mice and ntg littermates. Immunopositive cells were assessed in prefrontal cortex (top panel), hippocampus (middle panel), and midbrain (bottom panel). Scale bar: 50 μm, 40x magnification. CTX, cortex, CA1, CA1 region of hippocampus, MID, midbrain.

**Fig. 6.**
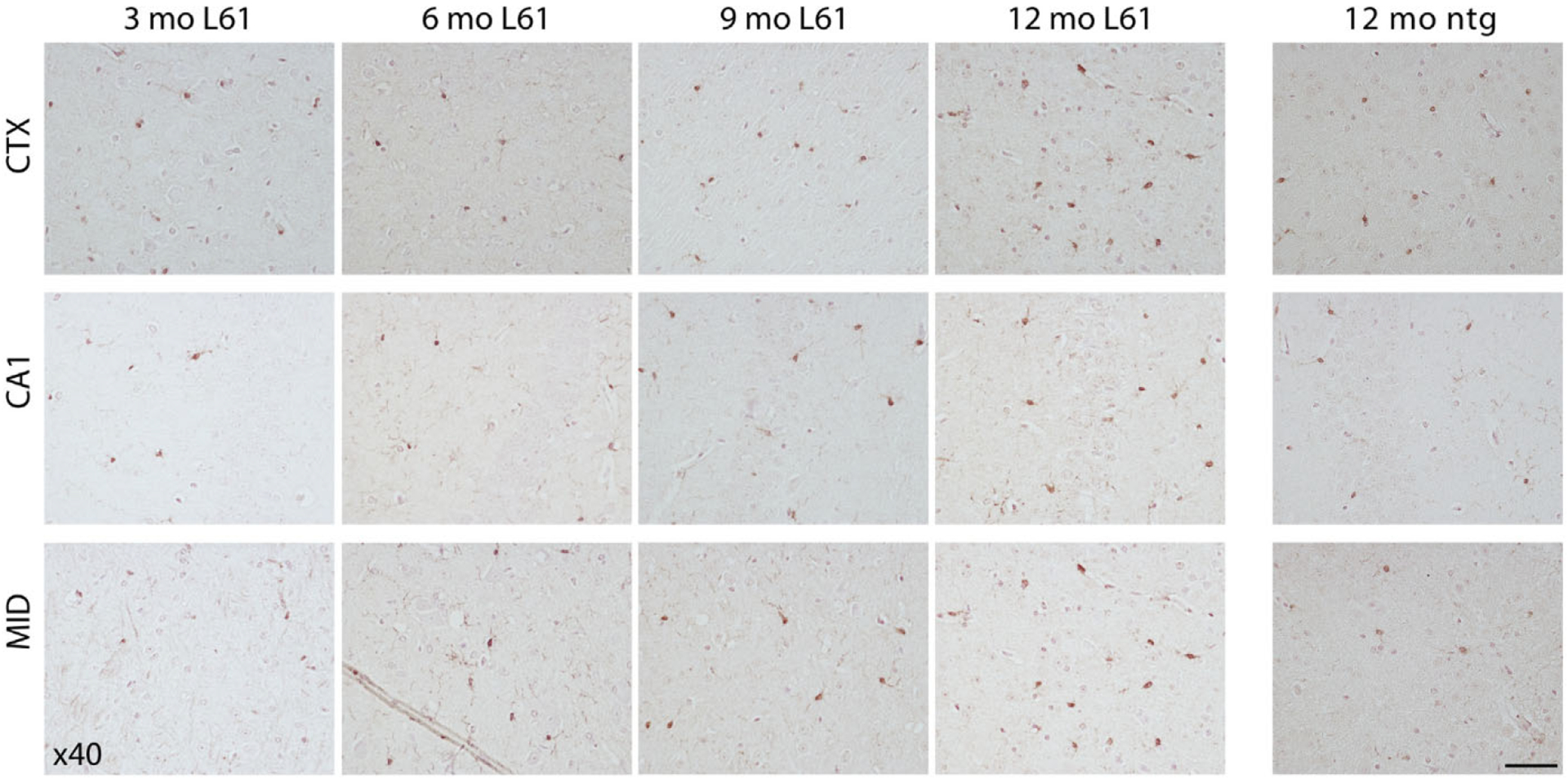
Activated microglia morphology and distribution in selected areas of the brain. Immunostaining with an antibody targeting ionized calcium binding adaptor molecule 1 (Iba1). Sections from 3-, 6-, 9-, and 12-month-old L61 tg male mice and ntg littermates. Immunopositive cells were assessed in prefrontal cortex (top panel), hippocampus (middle panel), and midbrain (bottom panel). Scale bar: 50 μm, 40x magnification. CTX, cortex, CA1, CA1 region of hippocampus, MID, midbrain.

IHC stainings with markers for astrocytosis (GFAP) and microgliosis (Iba1) were performed in areas with profound α-syn pathology, including hippocampus, prefrontal cortex, and midbrain (Figs. 5 and 6). We did not observe any global age-related abnormalities in the morphology or distribution of activated glial cells in the L61 tg mice or any such differences between L61 tg and ntg mice. Moreover, no clear sex-related differences were observed.

To further assess astrocyte activation, the levels of GFAP were measured by ELISA and compared between L61 tg mice and ntg age-matched controls. No differences in GFAP levels could be observed, neither between L61 tg and age-matched ntg control males, nor between different age groups of L61 males and females (F_(3,29)_ = 1.091, *p* = 0.368 and F_(3,25)_ = 2.348, *p* = 0.096, respectively) (Supplementary Fig. 2).

### 3.4. Behavioral phenotype and survival

**Fig. 7.**
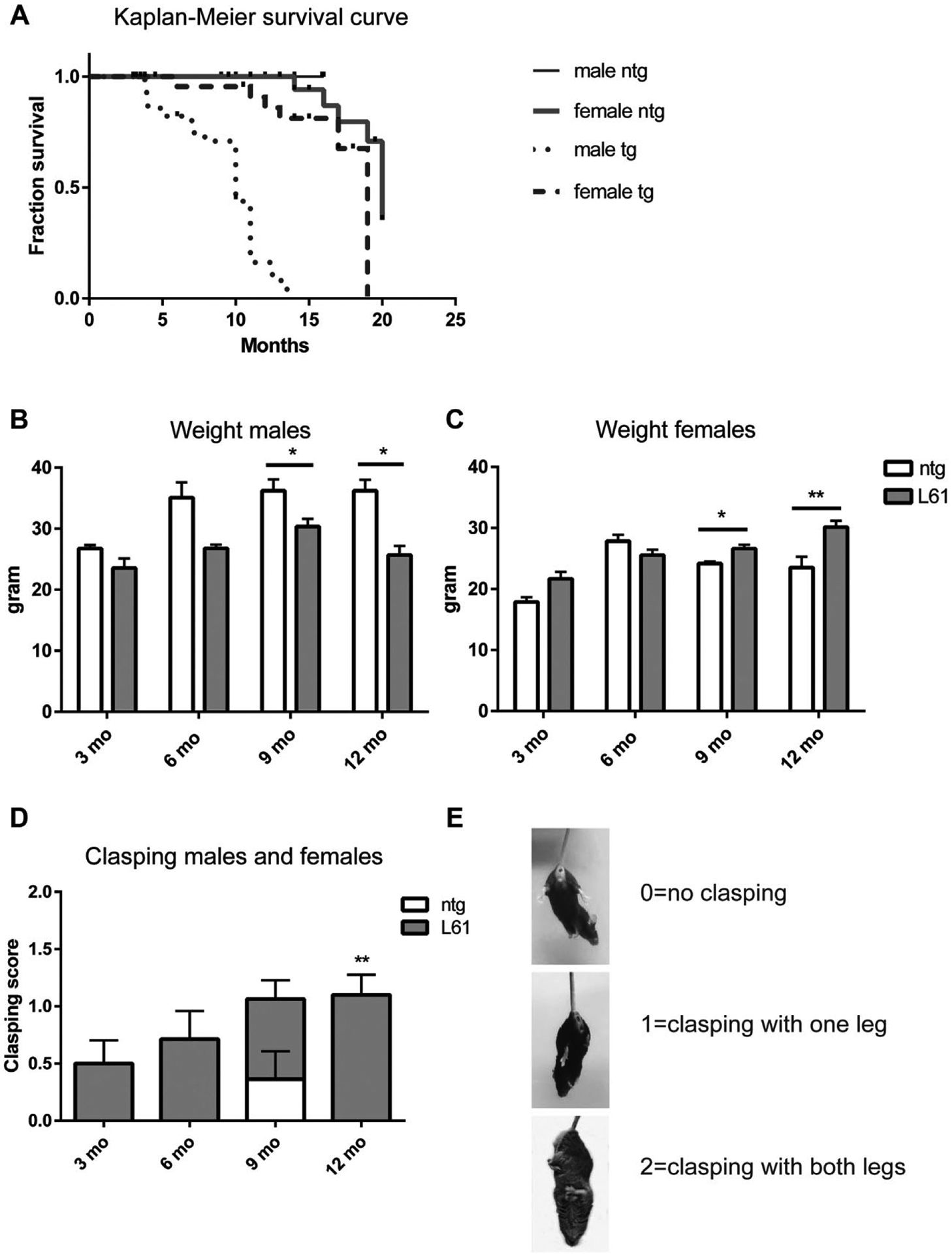
Lifespan, weight, and hind limb clasping behavior. Male L61 tg mice had a reduced life span from 4 months of age, whereas females lived almost as long as ntg control mice (A). Male L61 tg mice displayed a weight loss compared with nontransgenic (ntg) controls (B), whereas female L61 tg mice gained weight with increasing age (C) Both male and female L61 tg mice displayed a progressive increase in hind limb clasping, whereas ntg controls did not show this behavior in general (D). The scale used to analyze clasping is shown in (E). Data shown as mean ± SEM, *p < 0.05, **p < 0.01.

The breeding between C57B16/DBA2 males and heterozygous L61 females on a C57/B16/DBA background was normal and resulted in the expected number of tg and ntg offspring. Phenotype-related changes in survival of the mice up to 22 months of age were analyzed with Kaplan-Meier curves, demonstrating a progressive decrease in survival in L61 tg mice compared with ntg controls (Fig. 7A). This decrease was particularly pronounced in males, where the decline in survival rates started at 4 months of age with a 7% increase in mortality compared with age-matched ntg male mice. Survival was significantly decreased in L61 tg males in comparison with L61 tg females (*p* < 0.0001) and ntg males (*p* < 0.0001), as well as in L61 tg females compared with ntg females (*p* = 0.0163). There was no difference in survival between ntg males and females (*p* = 0.3553). The median survival (the age when 50% of the subjects had died) for L61 tg males was decreased compared with ntg males (10 months and >22 months which was the study end point, respectively), whereas there were no differences in median survival between L61 tg and ntg females (19 and 20 months, respectively). After 18 months, the proportion of surviving mice was 0% for L61 tg males, 62.6% for L61 tg females, 79.7% for ntg females, and 100% for ntg males.

From 6 months of age, L61 tg male mice started to display weight loss, and at 9 months of age, there were significant differences between these mice and age- and sex-matched ntg controls (Fig. 7B). In contrast, L61 tg females displayed a higher weight than ntg controls from 9 months of age (Fig. 7C).

### 3.5. L61 tg mice develop age progressive hind limb clasping and hyperactivity

Mice were assessed for visible hind limb clasping and locomotor activity. Neither male nor female L61 tg mice showed any significant age progression of such motor impairments. However, male L61 tg mice displayed motor impairments more often compared with females at 12 months (Kruskal-Wallis ANOVA *p* = 0.0001, H = 14.5 followed by Mann-Whitney *U* test, *p* = 0.013, U = 20.5; Z = 2.39 at 12 months). None of the ntg controls showed any sign of overt motor impairments at any of the analyzed time points.

The L61 tg mice displayed an age-related increase in hind limb clasping compared with ntg controls. Some L61 tg mice displayed clasping behavior already at 3 months of age and clasping progressed with age (Fig. 7D). Significant differences between L61 tg and controls were observed at 12 months of age (Mann-Whitney *U* test, *p* = 0.0068, U = 15, Z = −2.71). There were no differences in hind limb clasping between male and female L61 tg mice. The hind limb clasping score scale used (0 = no clasping, 1 = clasping with one hind leg, and 2 = clasping with both hind legs) is shown in Fig. 7E.

**Fig. 8.**
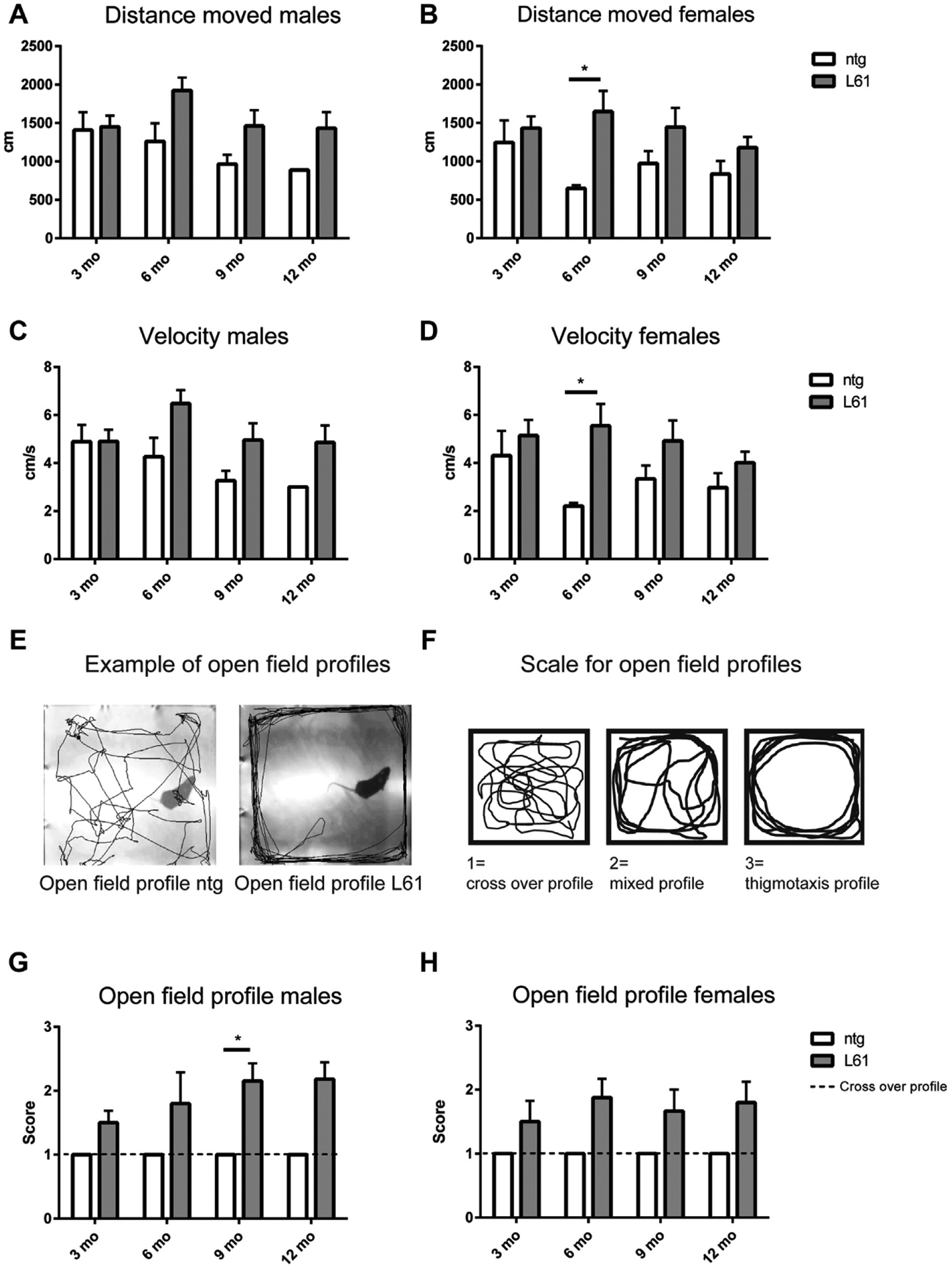
Open field test. Both male and female L61 tg mice displayed a hyperactive phenotype, including a longer distance moved (cm) (A, B) and a higher velocity (cm/s) (C, D), than nontransgenic (ntg) controls from 6 months of age. The tracks from the open field test showed that the movement pattern differed between ntg and L61 tg mice (E). The scale used for scoring the different profiles is shown in (F). All ntg mice (males and females) showed a normal cross over profile in the open field test, whereas L61 tg mice showed an increased score, indicating a thigmotaxis movement pattern (G, H). Data shown as mean ± SEM, *p < 0.05.

In the open field test, both male and female L61 tg mice manifested a hyperactive phenotype, including increased distance moved and velocity at 6 months of age. However, when looking at sex differences in different age groups, significant differences were only observed for females (Kruskal-Wallis ANOVA, *p* = 0.004, H = 8.3 (distance moved) and (*p* = 0.0065, H = 7.41 (velocity) followed by Mann-Whitney *U* test, *p* = 0.044, U = 0, Z = −1.96 for both variables) (Fig. 8A–D). After 6 months of age, the activity decreased for both L61 tg mice and ntg controls. Several of the L61 tg mice displayed a thigmotaxis profile, running around close to the walls of the open field, which suggests an anxious phenotype. In contrast, all ntg mice displayed a normal crossover profile as shown in the examples of tracks that are given in Fig. 8E. All tracks from the open field test were scored according to the following scale: 1 = cross over, 2 = mixed, or 3 = thigmotaxis as shown in Fig. 8F. Both male and female ntg control mice were scored as having a cross over profile, meaning that they were exploring the whole open field arena. In contrast, the L61 tg males showed an age-dependent increase in thigmotaxis behavior (Kruskal-Wallis ANOVA, *p* = 0.0003, H = 13.34, followed by Mann-Whitney *U* test, *p* = 0.018 at 9 months of age). In addition, there was a trend toward an increase in thigmotaxis behavior in L61 tg females compared with ntg controls. Moreover, L61 tg mice showed a tendency to more wall rearing but less free rearing activity compared with ntg controls, which might be due to motor deficiencies (data included in the PCA described in the following).

**Fig. 9.**
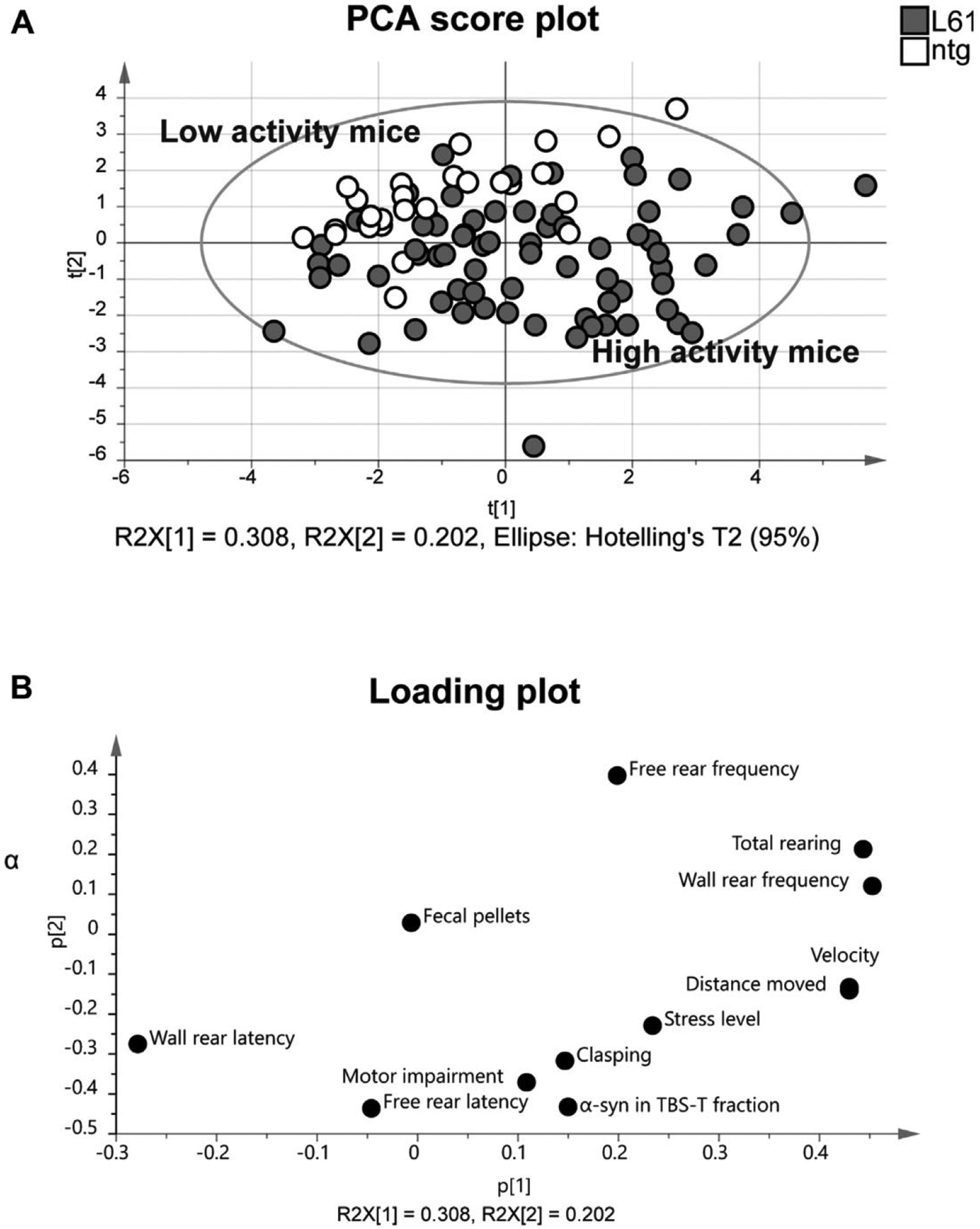
Principal component analysis (PCA) of the behavioral parameters and brain levels of soluble α-syn aggregates. All variables in the open field test and hind limb clasping behavior as well as the TBS-T soluble α-syn aggregate levels are integrated into the scores of the first 2 principal components (t1 and t2) for individual mice at all age groups. The L61 tg mice (gray) were mainly positioned to the right and lower parts of the score plot and were thus separated from the nontransgenic (ntg) controls (white) that were mainly positioned in the upper left part of the score plot (A). In general, the L61 tg mice were more active in the open field test and displayed higher hind limb clasping scores than ntg controls. All variables included in the PCA are shown in the loading plot (B).

To visualize possible group differences, PCA was used. All parameters from the open field test and hind limb clasping scores, as well as TBST-T soluble α-syn aggregates levels, showed a clear separation between L61 tg mice and ntg controls. The ntg control mice (male and females, all ages) were clustered in the upper left part of the score plot, based on all included variables, showing that they have a homogeneous profile. In contrast, the L61 tg mice (male and females, all ages combined) displayed a heterogeneity and were distributed in the score plot, mostly in the right upper and lower part (Fig. 9A). The loading plot shows all variables included in the analysis (Fig. 9B). The score plot and the loading plot are super-imposable and high-activity mice (e.g., L61 tg mice) are characterized by high velocity, long distance moved and more rearing activity in the open field test, higher hind limb clasping score, and higher stress levels. As a contrast, low-activity mice (i.e., ntg mice) displayed lower values of all these variables. The number of fecal pellets in the open field test did not differ between the groups. The number of urine spots was not included in the analysis because only few animals urinated in the arena.

### 3.6. Higher brain levels of α-syn in mice with motor impairments

**Fig. 10.**
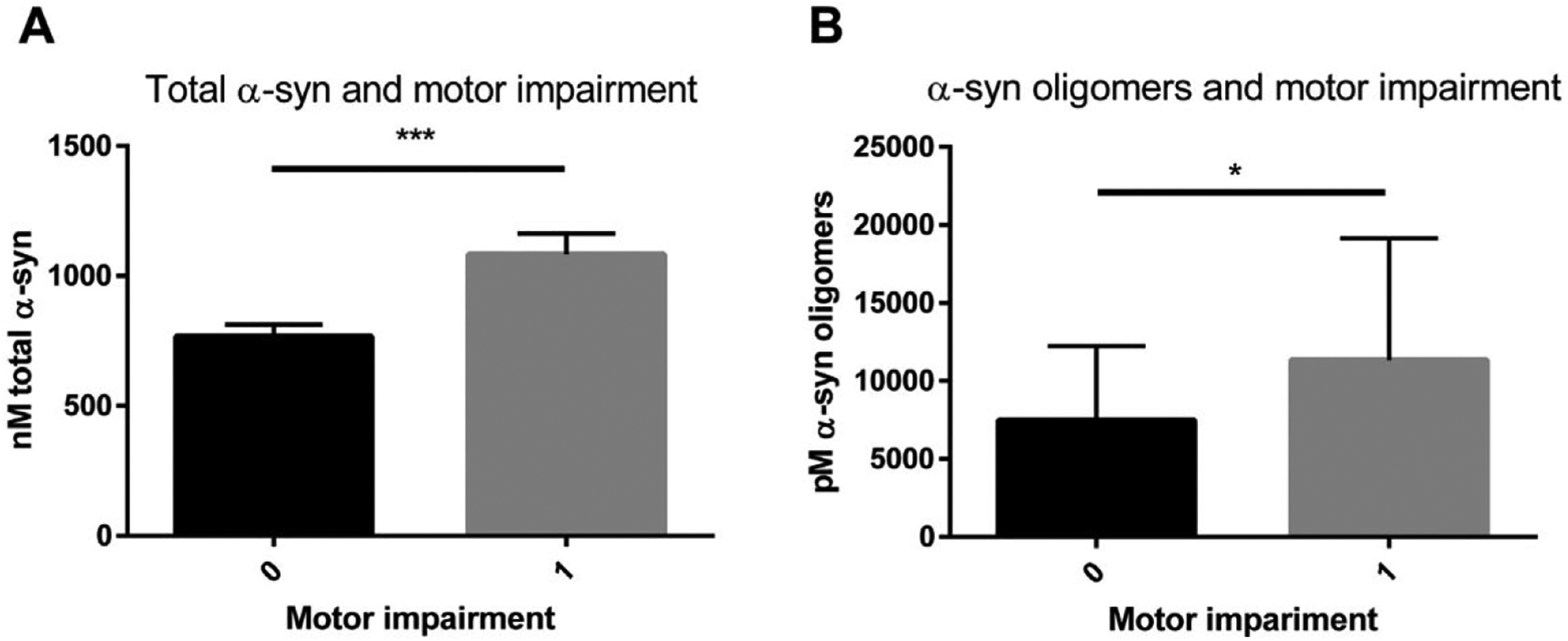
Analyses of total and TBS-T soluble aggregated α-syn in mice with and without motor symptoms. Both male and female L61 tg mice that displayed visible motor symptoms, including ataxia and gait problems, had higher levels of total α-syn (A) and α-syn oligomers (B) in the brain. 0 = no motor-impairment and 1 = visible motor-impairment. Data shown as mean ± SEM, *p < 0.05 and ***p < 0.001.

To analyze possible correlations between brain levels of α-syn and behavioral outcome, Spearman-rank correlation was used. No significant correlations were found between the TBS or TBS-T fractions of total and oligomeric α-syn levels, respectively, and behavioral variables (open field test and hind limb clasping). However, L61 tg mice that displayed overt motor impairments, as unsteady movement and staggering gait, showed higher levels of both total α-syn and TBS-T soluble aggregated α-syn levels in the brain (unpaired *t*-test, (*p* < 0.001 (Fig. 10A) and *p* = 0.014, respectively) (Fig. 10B). Among the animals that developed motor impairments, 83% were L61 tg males, which is consistent with higher mortality and more severe phenotype exhibited by L61 tg male mice than females. Further, the proportion of males with motor impairment in each age group was: 3 months 25%, 6 months 40%, 9 months 62%, and 12 months 73%. In contrast, the proportion of females with motor impairments was lower: 3 months 0%, 6 months 11%, 9 months 25%, and 12 months 10%.

## 4. Discussion

In this study, we found an age progressive increase in the levels of soluble α-syn aggregates in male and female L61 tg mice, as well as higher levels of total and aggregated species of α-syn in animals that display severe motor impairments. This study also adds a detailed analysis of mortality in this transgenic α-synucleinopathy mouse model.

### 4.1. Age-related changes in aggregated α-syn levels and α-syn deposits

The L61 tg mice have previously been shown to exhibit an age-dependent increase of total α-syn levels in specific brain regions (e.g., in the hippocampus, but not in the striatum) (Rabl & et al., 2017) of male mice. We did not observe any such age-related increase in total α-syn levels in male L61 tg mice. However, we analyzed the whole left hemisphere, whereas in the previous study (Rabl & et al., 2017) isolated tissues were subdissected before protein extraction. Thus, the presence of local age-related changes cannot be excluded.

The levels of different forms of aggregated α-syn in L61 mice of various ages have not been analyzed in previous studies. We performed a sequential extraction of α-syn in the brain samples, where we separated α-syn by solubility and aggregation state. The sequential extraction allows for a separation of the TBS and TBS-T soluble forms of α-syn from the insoluble, highly aggregated α-syn predominantly found in the FA fraction.

Although soluble α-syn aggregates are not easy to study in nondenaturing conditions, several ELISAs for this purpose have been developed (Paleologou & et al., 2009; Majbour & et al., 2016; Fagerqvist & et al., 2013; Brannstrom & et al., 2014; El-Agnaf & et al., 2006). Here, we used a sandwich ELISA to detect soluble α-syn aggregates in brain homogenates by using an antibody (MJFR14-6-4-2) that selectively binds soluble α-syn aggregates. By applying this antibody both for coating and detection, we further excluded the possibility that also monomeric α-syn would be measured. It has been shown that MJFR-14-6-4-2 specifically binds to an epitope on both filamentous and oligomeric α-syn. As this epitope can be detected both on diseased human brain tissue and in brains from α-syn tg mice (Sampson & et al., 2016), it should be considered relevant for PD studies.

We found that soluble α-syn brain aggregates were increased in the TBS-T fraction, containing less soluble aggregated α-syn, but not in the TBS fraction containing more soluble species, in aged L61 tg mice. In addition, the TBS-T/TBS ratio of aggregated α-syn showed a significant age progression. These data suggest that the α-syn aggregation process intensifies with aging. In line with our results, increased α-syn phosphorylation and oligomerization have been shown in aging cynomolgus monkeys (Chen & et al., 2016) as well as in a knockin α-syn mouse model (Ho & et al., 2020). Moreover, due to the fact that analyzed samples were extracted from the whole hemisphere, more profound local changes in soluble α-syn aggregates levels cannot be excluded. No age-related differences were observed in the levels of total α-syn in the FA fraction, although females showed lower levels of FA fraction α-syn than males. This, together with the evidence that the MJFR14-6-4-2 antibody selectively targets soluble α-syn aggregates even in samples that contain high levels of monomeric α-syn (Lassen & et al., 2018), further supports the notion that the increase in α-syn levels with age predominantly arises from an increase in the soluble, prefibrillar and oligomeric forms of α-syn. Our observations suggest that in L61 tg mice, especially in males, where brain levels of α-syn (in both TBS, TBS-T, and FA fractions) are high from a young age (3 months), the main mechanism underlying the progression of α-syn pathology might be aggregation rather than a general increase of protein levels.

Even though both male and female L61 tg mice show a robust behavioral phenotype, we could not correlate individual behavioral variables with levels of α-syn in the brain. Both male and female L61 tg mice develop overt motor impairments as end-stage symptoms, including gait problems and ataxia. Within weeks after the onset of such symptoms, mice had to be euthanized due to the severity of such disturbances. Both the levels of total α-syn and soluble α-syn aggregates were higher in animals that suffered from severe motor symptoms. However, only TBS-T soluble α-syn oligomers showed an age progressive increase suggesting that an increased presence of aggregated α-syn may be a crucial factor for the pathologic development in these mice. This is in line with our previous findings in (Thy-l)-h[A30P] mice, where increased levels of α-syn oligomers also were related to motor symptoms (Lindstrom & et al., 2014).

Consistent with previous studies (Rabl & et al., 2017), we observed high immunoreactivity of p129 α-syn. In PD patients, the deposited Lewy bodies in the brain mainly consist of p129 α-syn (Anderson & et al., 2006; Fujiwara & et al., 2002) and it has been suggested that these species of α-syn might be crucial in the spreading and progression of pathology (Karampetsou & et al., 2017). In contrast to α-syn oligomers, which can be accurately measured with ELISA, there are controversies related to the accuracy of detecting p129 α-syn on brain sections (Waxman et al., 2008). Therefore, in our IHC experiments, we used antibodies against p129 α-syn (the phospho-specific clone EP1536Y (Delic & et al., 2018)) as well as human α-syn (clone 211) to assess overall α-syn pathology. Because we analyzed regional p129 α-syn pathology, these IHC-based results did not closely reflect the age progressive increase in p129 α-syn levels, as observed in the ELISA-based data on whole hemisphere preparations. L61 tg mice showed high levels of phosphorylated and nonphosphorylated α-syn in various areas of the brain, including substantia nigra, striatum, neocortex, and hippocampus. In addition, we observed that p129 α-syn accumulated in neuronal cell bodies, whereas nonphosphorylated α-syn was also found in nerve terminals, in line with the proposed physiological role of α-syn in synaptic vesicle system regulation (Burre, 2015; Almandoz-Gil & et al., 2018).

### 4.2. Neuroinflammation

During the immune response, astrocytes undergo morphologic and physiological changes, mainly hypertrophy of cellular processes and upregulation of GFAP (Brahmachari et al., 2006). While shifting from a quiescent to an activated state Iba1-positive microglial cells also undergo profound morphologic changes. Resting microglia have long ramified processes with small cell bodies, whereas their activation induce remodeling leading to enlargement of ameboid shape cell bodies and shortening of processes (Pekny et al., 2014; Bruck & et al., 2016).

In addition to the neuronal Lewy bodies, α-syn can accumulate in astrocytes of aging (9–10 months old) L61 tg mice (Kim & et al., 2018). Moreover, in vitro studies indicate that astrocytes containing intracellular deposits of aggregated α-syn can contribute to cell-to-cell spreading of α-syn pathology (Rostami & et al., 2017). Previous studies have shown signs of neuroinflammation in L61 tg mice, such as age-related elevation in GFAP and Iba1 levels in isolated brain structures, including neocortex, striatum, and hippocampus of 6 and 10 months old L61 tg mice (Rockenstein & et al., 2002; Price & et al., 2018; Kim & et al., 2015).

As we did not observe any abundant presence of GFAP and Iba1 positive cells in relevant brain areas, we can exclude the presence of global inflammatory responses. However, because of our extraction strategy, we cannot exclude a more local reactivity. This would be in line with a previous report, showing that the α-syn burden does not cause global activation of glial cells, but rather local changes in microglial phenotype, in these mice (Watson & et al., 2012). In addition, it was found that only microglial cells located in striatum and substantia nigra of aging L61 tg mice have larger cell bodies (which indicates a switch from a quiescent to an activated state), whereas in cerebral cortex hyper-ramified microglial cells were observed (Watson & et al., 2012).

Taken together, these findings suggest that the expression level of glial markers might not directly imply the activation status of the cells and measuring the levels of such markers is not a useful strategy to pinpoint the glial response to α-syn burden in L61 mice. Future studies should be focused on analyzing local changes in astrocyte and microglia reactivity.

### 4.3. Animal health and survival

In this study, we have characterized biochemical and behavioral differences between male and female L61 tg mice and their ntg littermates, from 3 up to 12 months of age. Our data indicate that L61 tg males lose weight during aging compared with ntg controls, whereas female L61 tg mice in contrast gain weight. The L61 tg mice have previously been reported to show a lack of increased mortality or morbidity until 14 months of age (Chesselet & et al., 2012). Based on our L61 tg breeding data, including 200 mice, the survival of males was reduced already at 4 months of age and their median survival was 10 months. Therefore, there is a risk that the data based on the oldest L61 tg males are biased as healthier mice, that are expected to have lower levels of α-syn pathology and a less severe phenotype, survived long enough to be included in the oldest age group. The differences in survival between laboratories may be explained by differences in housing conditions and in genetic backgrounds, contributing to the development of a more severe phenotype in our study. Such differences may for example result in differences in the gut microbiome and it was recently showed that these factors can affect the expression of motor deficits, microglial activation, and α-synuclein pathology in the L61 tg mouse model (Sampson & et al., 2016).

### 4.4. Development of behavioral impairments

Previous studies with L61 tg mice have indicated behavioral impairments at different stages (Chesselet & et al., 2012). For example, muscle strength and coordination in the wire hanging test and nest building skills are reduced already at 1 month of age (Rabl & et al., 2017). This is followed by increased latency to fall off the rotarod and fine motor impairments in the challenging beam test (Rabl et al., 2017; Chesselet & et al., 2012; Fleming & et al., 2004). In the present study, mice were assessed from 3 months of age, after the onset of fine motor impairments but before the onset of overt motor symptoms. Neither male nor female L61 tg mice showed any sign of visible gait problems or ataxia when included in the study. We used hind limb clasping as a marker for disease progression, and clasping scores were increased in the older age groups of L61 tg mice. Sex did not affect hind limb clasping, indicating that also female L61 tg mice show a progressive dysfunction in these behavioral measures.

Both male and female L61 tg mice showed a hyperactive phenotype at 6 months of age, with longer distance moved and higher velocity in the open field test. Previous studies have shown that L61 tg mice are less active in the open field test at 14 months of age and that this behavior is preceded by hyperactivity at 4–5 months of age, with increased distance moved and higher velocity (Chesselet & et al., 2012). The early hyperactive behavior has been related to changes in dopamine release and increases in extracellular dopamine that precede the observed loss of striatal dopamine at 14 months of age (Lam & et al., 2011). These observations are consistent with our results, which show that L61 tg mice at 6 months of age are more active than ntg controls, but that the activity after this time point starts to decline. In addition, the L61 tg mice showed a deviant behavioral profile when exploring the open field arena, with increased thigmotaxis (moving in circles close to the wall of the open field arena) compared with ntg mice.

The PCA, including behavioral and biochemical data from males and females at all age groups, showed that L61 tg mice are scored as more active compared with their ntg littermates. However, no clear grouping of males and females or different age groups was observed in the PCA score plot. Previous studies have shown that young L61 tg mice (4–5 months of age) suffer from gut dysfunction, resulting in a reduction in fecal pellet output (Wang & et al., 2008). However, we could not observe any differences in the amount of fecal pellets in the open field test, as shown in the loading plot of the PCA, which might be a consequence of a shorter time spent in the arena.

### 4.5. Implementation of the model

Despite ongoing efforts to limit the number of animals in pre-clinical studies, there is still a need to use disease models that accurately reflect various aspects of α-synucleinopathies. Although prevalence and progression show sex-related differences in PD and other neurodegenerative disorders, most of the reported studies have only included animals with the same sex, mainly males. Therefore, it is of utmost importance to consider sex-specific features of the animal models (Lee, 2018). As female L61 tg mice display increased survival rate, lower levels of α-syn and a milder behavioral phenotype than male mice, they could be said to more accurately reflect features of sporadic PD and should thus be better suited for long-term treatment studies. Male mice, on the other hand, with their severe and fast progressing pathology together with a shortened lifespan, may be better suited for short-term proof-of-concept studies aimed at more acutely reducing α-syn aggregation and mortality.

## 5. Conclusion

To conclude, we have observed an age-related shift toward more aggregated forms of α-syn in the brain of L61 tg mice. Importantly, the levels of total and oligomeric α-syn were significantly higher in animals that displayed severe motor impairments at the end point of the experiment, regardless of age. This finding suggests a connection between α-syn aggregation and development/severity of motor impairments. Thus, the L61 tg mouse model should be a suitable tool for studies of α-syn oligomer—induced toxicity and evaluation of new therapies specifically targeting α-syn oligomers.

## Supplementary Material

Figure 1

Figure 2

## Figures and Tables

**Table 1:** Coating and detection antibodies used for the respective ELISAs

Assay	Oligomeric α-synuclein	Monomeric α-synuclein	GFAP
coating	MJFR-14-6-4-2 (Abcam, ab209538) 1 μg/mL	MJFR1 (Abcam, ab138501) 0.25 μg/mL	mouse-anti GFAP (Sigma Aldrich, G3893) 0.5 μg/mL
detection	MJFR-14-6-4-2 biotinylated (Abcam, ab209538) 1 μg/mL	Syn1 (BD Biosciences, 610787) 0.35 μg/mL	rabbit-anti GFAP (Dako, Z0334) 0.96 μg/mL
HRP	Streptavidin (Mabtech, 3310-9) 1:2000	anti-mouse IgG F (ab’)_2_ (Jackson Immuno Research, 115-036-006) 0.4 μg/mL	anti-rabbit IgG (H + L) (ThermoFisher, 31,460) 0.4 μg/mL

HRP, horseradish peroxidase.
