# Supplementary figures and images for: Age-related increase of alpha-synuclein oligomers is associated with motor disturbances in L61 transgenic mice
Source: Neurobiol Aging. Author manuscript; Available in PMC 2022 Nov 10. (PMC9648497; doi:10.1016/j.neurobiolaging.2021.01.010)

### Sequential extraction of $\alpha$ -synuclein

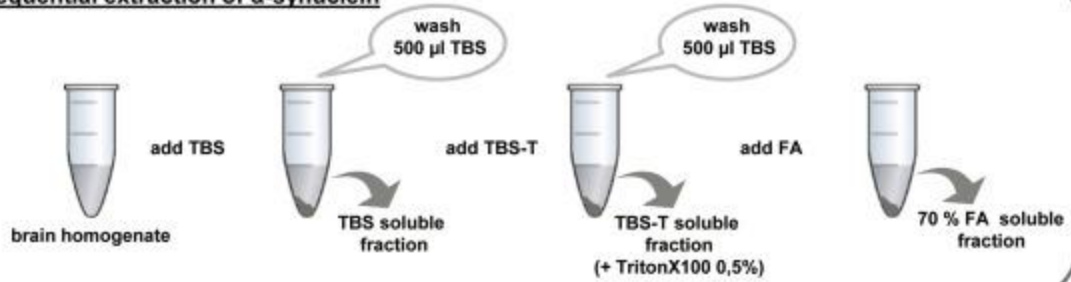

Supplement: Figure 1 [file NIHMS1795431-supplement-Figure_1.pdf]

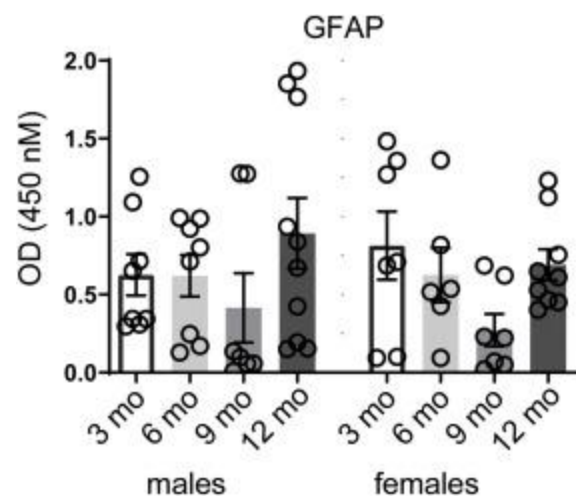

Supplement: Figure 2 [file NIHMS1795431-supplement-Figure_2.pdf]
